# In Situ EXAFS Study of Mo–P Bond Dynamics in Molybdenum Phosphide during CO_2_ Reduction and Hydrogen Evolution

**DOI:** 10.1021/acsomega.6c02246

**Published:** 2026-05-15

**Authors:** Karen A. Castañeda, Ishani Senevirathna, Benard Patawah, Ning Su, Mohammad Asadi, Carlo U. Segre

**Affiliations:** † Department of Physics, 2455 Illinois Institute of Technology, 3101 South Dearborn Street, Chicago, Illinois 60616, United States; ‡ Center for Synchrotron Radiation Research and Instrumentation, 3101 South Dearborn Street, Chicago, Illinois 60616, United States; § Department of Chemical and Biological Engineering, Illinois Institute of Technology, 10 West 33rd Street, Chicago, Illinois 60616, United States; ∥ Department of Mechanical, Materials and Aerospace Engineering, Illinois Institute of Technology, 10 West 33rd Street, Chicago, Illinois 60616, United States

## Abstract

Transition Metal Phosphides (TMPs) are widely studied as catalysts in reactions like hydrogen evolution, oxygen reduction, and hydrodesulfurization. TMPs have demonstrated remarkable efficiency in accelerating reactions by providing active sites for adsorption and facilitating the transformation of reaction intermediates, a feature that has increased the interest of TMPs in the electrochemical carbon dioxide reduction reaction (eCO_2_RR). Despite their growing use, the potential-dependent structural behavior of TMPs under operating electrochemical conditions remains insufficiently understood. This work investigates the dynamics of the Mo–P bond during eCO_2_RR and hydrogen evolution reaction (HER) using in situ extended X-ray absorption spectroscopy (EXAFS) studies at the Mo–K edge. Structural characterization of the synthesized MoP nanoparticles was performed by powder X-ray diffraction (XRD) and transmission electron microscopy (TEM). Results of the overall electrochemical activity of MoP are presented through linear sweep voltammetry (LSV) experiments in the potential range of 0 to −1.5 V versus Ag/AgCl and chronoamperometry (CA). Liquid products were analyzed by ^1^H NMR spectroscopy. The synthesized catalyst showed increasing activity with the highest Faradaic efficiency of 11.75% for C_2+_ products at −1.3 V vs Ag/AgCl. In situ EXAFS revealed a change in the average Mo–P bond distance during eCO_2_RR, whereas no such change was observed during the hydrogen evolution reaction (HER). This finding suggests that phosphorus, particularly through modulation of the Mo–P bond length, plays an important role in the eCO_2_RR and demonstrates how in situ EXAFS can track local structural perturbations in MoP catalysts during electrochemical operation, providing complementary structural insight that correlates with electrochemical behavior.

## Graphical abstract

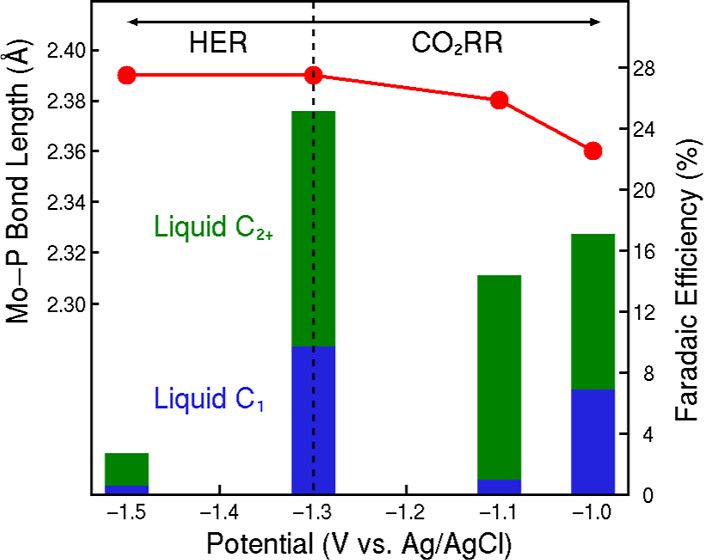

## Introduction

As the global CO_2_ concentration in the atmosphere reaches elevated levels, increasing the greenhouse effect, it is necessary to find effective ways to reduce these emissions, one of which is the reduction of CO_2_ into valuable fuels using electrocatalytic methods. In this context, transition metal phosphides (TMPs) are considered promising catalysts for the electrochemical CO_2_ reduction reaction (eCO_2_RR).^1^

In a recent study, Cu_3_P was used as a catalyst for reducing CO_2_ into formate with a maximum Faradaic efficiency (FE) of 8%, but with no reported C_2+_ formation.^2^ The limited selectivity observed highlights the challenge of suppressing the competing hydrogen evolution reaction (HER).

Another study used nickel phosphides (Ni_2_P, Ni_12_P_5_, Ni_5_P_4_, and NiP_2_) to electrochemically reduce CO_2_ to methylglyoxal and 2,3-furandiol, reaching a maximum FE of 71% for 2,3-furandiol.^3^ The study establishes a structure–composition relationship, where increasing phosphorus content in the nickel phosphide correlates with enhanced selectivity toward multicarbon oxygenates. However, no in situ experiments were performed to directly verify these mechanistic changes.

In parallel, density functional theory (DFT) calculations on 29 transition metal phosphides have shown that only six (CrP, HfP, TaP, TiP, VP, and ZrP) are suitable for CO_2_RR. In these systems, the reaction pathway proceeds through the *OCHO intermediate, which is more favorable than both *COOH formation and hydrogen adsorption. The studied catalysts were found to produce formic acid, methanediol, and methanol.^4^

A recent study from our research group reported imidazolium-functionalized ionomer-confined molybdenum phosphide (MoP) as an effective catalyst for eCO_2_RR. In this system, imidazolium enhances CO_2_ diffusion (mass transport) to the catalyst surface and tunes the adsorption energy of the *CO intermediate, thereby reducing the required overpotential.^5^ These improvements have been attributed to modifications of the catalyst microenvironment, which increase CO_2_ availability and partially suppress the hydrogen evolution reaction (HER), as supported by in situ Raman spectroscopy.

In a related study from our research group, imidazolium-functionalized Mo_3_P achieved high selectivity toward C_3_ products, with Faradaic efficiencies up to 91%, further highlighting the role of microenvironment engineering in promoting multicarbon product formation.^6^ These findings suggest that controlling the catalyst microenvironment can shift the competition between eCO_2_RR and HER toward CO_2_ reduction; however, the underlying structural changes at the atomic level that govern this selectivity remain unclear.

In all these cases, achieving high selectivity toward C_2+_ products remains a central challenge in eCO_2_RR, particularly due to the competing hydrogen evolution reaction (HER) in aqueous media.^7,8^

The well-documented catalyst MoP,^9–11^ which finds use in everything from hydrotreating reactions to electrochemical hydrogen evolution^12–14^ has not yet been analyzed from a more fundamental perspective, especially in terms of its potential-dependent structural evolution and the mechanisms that differentiate HER and eCO_2_RR.

Although in situ Raman spectroscopy has been widely used in eCO_2_RR studies to probe the local reaction environment, it provides indirect information about the catalyst structure. In contrast, in situ EXAFS offers direct insight into changes in coordination environment and bonding under operating conditions, making it a powerful complementary technique for understanding catalyst behavior at the atomic scale.

With this in mind, we have prepared MoP and investigated its local structural changes by extended X-ray absorption fine structure (EXAFS), as a function of applied potential in an alkaline electrolyte under both eCO_2_RR and HER conditions. This work highlights the importance of in situ structural characterization for understanding TMP electrocatalysts’ role under realistic reaction conditions.

## Materials and Methods

(NH_4_)_6_ Mo_7_O_24_ ·4H_2_O (Sigma-Aldrich, BioUltra), (NH_4_)_2_ HPO_4_ (Sigma-Aldrich, ACS reagent), and C_6_H_8_O_7_ (Sigma-Aldrich, ACS reagent) as a chelating agent were dissolved in 50 mL of DI water at 90 °C for 12 h. The molar ratio was adjusted to Mo/P/CA = 1:1:2. After most of the water had evaporated, the remaining slurry was dried in a vacuum oven at 120 °C for 24 h and ground by hand. The precursor was then calcined at 500 °C for 5 h under Ar flow to obtain the precursor material, which was subsequently treated at 350 °C for 0.5 h and 700 °C for 2 h under flowing 5% Ar/95% H_2_ atmosphere at a flow rate of 0.5 L/min. All heating steps were performed at a constant rate of 5 °C/min.

Powder X-ray diffraction (XRD, Bruker D2 Phaser) was used to identify the phase purity and crystallinity of the studied catalyst. The XRD pattern was obtained using Ni-filtered Cu Kα radiation and a 192 channel Lynxeye position sensitive detector from 10° to 80° 2θ with a step size of 0.01° and counting time of 0.5 s/step. The resulting powder diffraction pattern was fitted by Rietveld refinement with GSAS-II^15^ using a nine-term Chebyshev polynomial function for the background. Additionally, images from a field-emission transmission electron microscope (JEOL JEM-2100F) were used to estimate the particle size distribution and morphology of the synthesized catalyst.

### Electrochemical Studies

All the electrochemical experiments were conducted using a modified H-type electrolytic cell. The electroreduction of CO_2_ was achieved in a two-compartment cell with a three-electrode system, which was separated by an anion exchange membrane (AEM, Dioxide Materials’ Sustaintion), and each compartment contained 40 mL aqueous 1 M KOH solution. The reference and counter electrodes were Ag/AgCl and Pt gauze, respectively. CO_2_ was delivered into the cathode compartment for at least 40 min before measurement at room temperature and under atmospheric pressure. During the experiments, CO_2_ and Ar were continuously delivered into the cathodic compartment for eCO_2_RR and HER, respectively. All potentials are presented relative to Ag/AgCl.

To further analyze the product distribution of CO_2_ reduction and assess the selectivity of MoP, chronoamperometric (CA) experiments were carried out at various constant potentials ranging from −1.0 to −1.5 V for 1 h (See Figure S1). Postexperiment, the liquid products were analyzed using nuclear magnetic resonance (Bruker Avance III 500 MHz) spectroscopy, 64 scans were accumulated with relaxation delays (d1) of 5s. The ^1^H spectrum was obtained using water suppression through a presaturation technique. NMR tubes were prepared by adding: 40 μL D_2_O, 50 μL of an 0.8 mM internal reference solution (DMSO), and 410 μL of the investigated solution, totaling 0.5 mL sample in each tube (See NMR spectra in Figure S2).

### Extended X-Ray Absorption Fine Structure Analysis

To investigate the electrochemical reaction mechanism behind the performance of MoP in the eCO_2_RR, in situ EXAFS spectra were taken on the MoP in fluorescence mode using a 5 grid fluoresence ion chamber at the Materials Research Collaborative Access Team (MRCAT) Sector 10, insertion device beamline at Argonne National Laboratory’s Advanced Photon Source.^16^ This beamline uses a liquid N_2_-cooled Si (111) double crystal monochromator followed by a harmonic rejection mirror to continuously scan the incident photon energy from ∼200 eV below to ∼700 eV above the Mo K-edge absorption energy of 20 keV. A molybdenum metal foil was used as a reference for energy calibration.

To carry out in situ XAS experiments, we ran CA tests in a window of potential highlighted in Figure 4. Twenty scans were taken and merged for each potential: −1.0, −1.1, −1.2, −1.3, −1.5 V vs Ag/AgCl (See Figure S3). The measurements were carried out on two electrolytes using a three-electrode custom-made cell; bare glassy carbon was used as the counter electrode and Ag/AgCl as a reference electrode; one electrolyte was CO_2_-saturated 1 M KOH and the second was 1 M KOH with Ar saturation. Figure 1 shows the in situ EXAFS cell used. EXAFS data were taken during chronoamperometric measurements, with each potential applied for 20 min. Ink consisting of 40 wt % carbon black, 20 wt % PVDF, 40 wt % sample, and 2 mL methylpyrrolidinone was used to deposit the MoP catalyst on the glassy carbon used as the working electrode.

**Figure 1:**
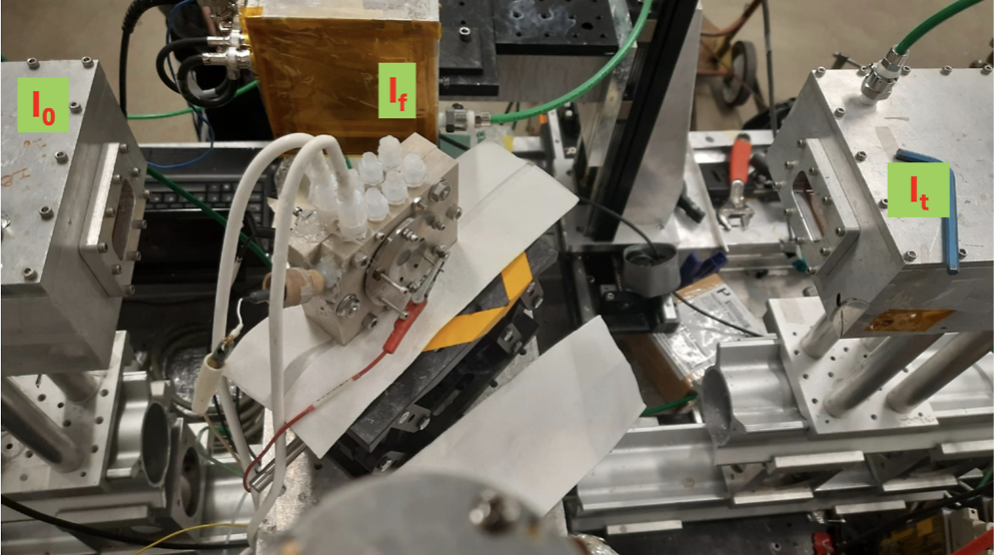
Custom cell used for in situ XAS experiments, showing the incident beam (*I*_0_), fluorescence detector (*I*_*f*_), and transmission detector (*I*_*t*_) used to monitor structural changes of the catalyst under operating conditions.

The EXAFS data was processed and fitted using the Larch software package,^17^ (See Supporting Information, Figures S4 and S5). The fits were performed with a k-weight of 2, using Hanning windows 2 Å^–1^ < *k* < 8 Å^–1^, dk = 2 and 1.0 Å < *R* < 2.5 Å using the 6-fold degenerate, nearest neighbor, single-scattering Mo–P path derived by FEFF8L from the ICSD-644084 crystallographic information file. The amplitude reduction factor (*S*_0_^2^ = 0.61) was extracted from the fit of the dry MoP nanoparticles, assuming full occupancy of the MoP_6_ octahedron. Using this amplitude reduction factor each data set was fitted independently with the result that the Mo–P path degeneracy was ∼6 and all values of Δ*E*_0_ were the same within their estimated standard deviations. The final combined fits were therefore performed with common Mo–P path degeneracy and Δ*E*_0_ parameters in order to minimize the estimated standard deviations of the variable parameters for each data set.

## Results & Discussion

### Structural Characterization

The XRD pattern in Figure 2 shows well-defined diffraction peaks corresponding to MoP, which match the International Crystal Structure Database (ICSD, Version 5.4.0) record number ICSD-644084, indicating the successful formation of a pure MoP phase with no detectable impurities. Structure refinement using GSAS-II resulted in hexagonal lattice parameters of *a* = 3.218(4) Å and *c* = 3.209(2) Å with isotropic size broadening consistent with a crystallite size of 7.3 ± 0.1 nm. The MoP TEM images in Figure 3 show that the MoP nanoparticles exhibit a spherical morphology and are well dispersed on a carbon matrix, likely derived from the pyrolysis of citric acid. The dark contrast regions observed in the pictures correspond to MoP particles, while the lighter background represents the supporting carbon. The scale bar of 50 nm confirms that the nanoparticles are uniformly distributed with minimal agglomeration. The particle size distribution histogram indicates that the MoP nanoparticles have an average diameter of approximately 22.5 nm, with most particles falling within the 15–30 nm range, suggesting a relatively narrow and uniform size distribution and limited crystallite aggregation.

**Figure 2:**
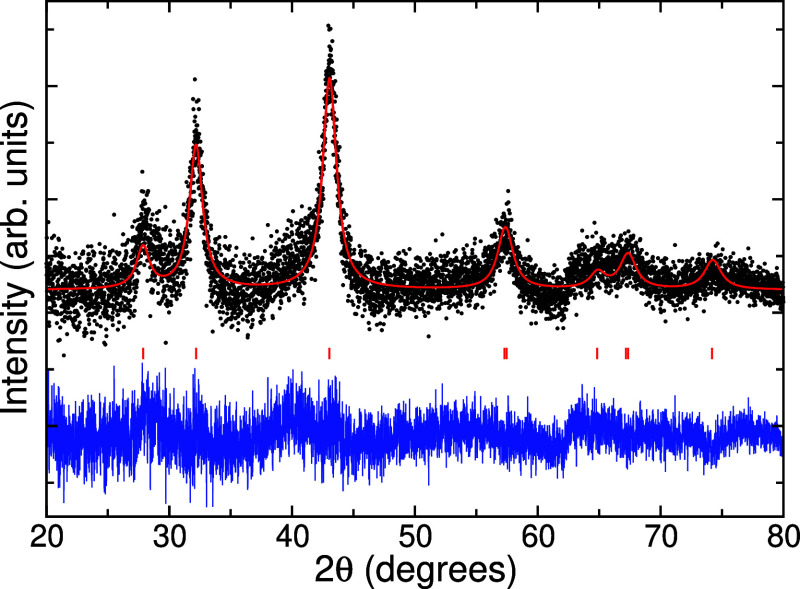
Powder X-ray diffraction (XRD) pattern of synthesized MoP (black), Rietveld fit with reflection tick marks (red), and difference (blue).

**Figure 3:**
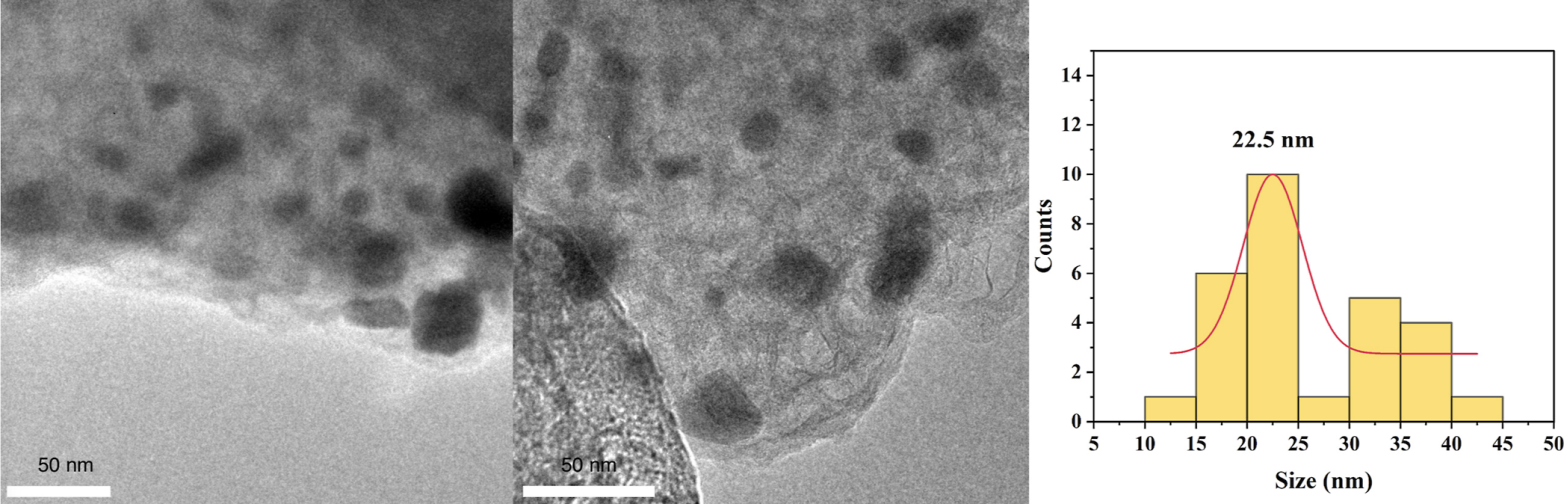
TEM images of the MoP nanoparticles and corresponding size distribution (average size: 22.5 nm). TEM image courtesy of Yuchen Zhang. Copyright 2025.

### Electrochemical Characterization

Figure 4 presents linear sweep voltammetry (LSV) measurements taken with a scan rate of 20 mV/s for MoP with and without CO_2_ saturation. These LSVs indicate that the eCO_2_RR and HER reactions compete for active sites. For eCO_2_RR, potentials beyond −1.3 V vs Ag/AgCl show that the strong selectivity for H_2_ generation of MoP dominates. For the HER scenario the overpotential required to drive the reaction is around −1.15 V. This is demonstrated by an increase in the overpotential required to drive the HER under CO_2_RR testing conditions. The change in HER activity and overpotential has been attributed to a variation in the species that donate protons; under acidic pH environments, hydronium donates protons, whereas in the near-neutral pH presented in CO_2_RR conditions, bicarbonate and water act as possible donors of protons for the HER.^7^

**Figure 4:**
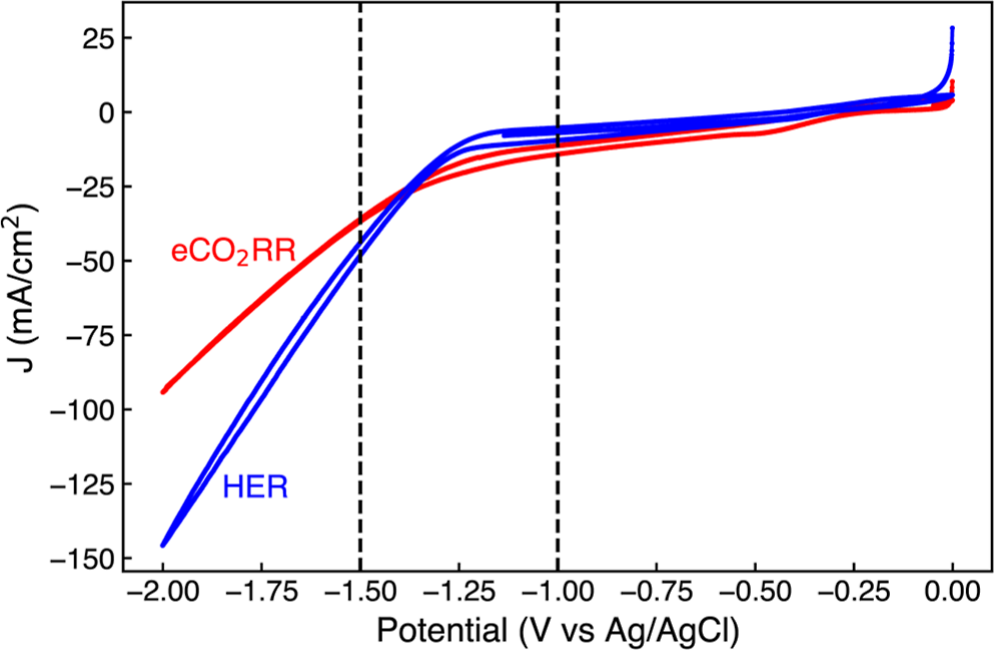
Linear sweep voltammetry (LSV) curves comparing MoP activity under CO_2_ (red) and Ar (blue) saturated conditions. The vertical lines define the potential window studied using EXAFS.

Figure 5 shows the Faradaic efficiency (FE) results for liquid products of the synthesized MoP NPs at cathodic potentials from −1.0 to −1.5 V vs Ag/AgCl, (See Appendix). The results indicate the presence of ethanol at the lowest potential (−1.0 V) with the highest 5.37% Faradaic efficiency for an applied potential of −1.3 V. The FE of acetate also reaches its highest value of 6.38% at −1.3 V. For the −1.5 V potential, alcohol production decreases and both acetate and formate production have close to 0.5% FE, suggesting that HER dominates at this potential. Although gaseous products were not quantified in the present study, prior work performed in our research group by Esmaeilirad et al.,^5^ demonstrated that MoP-based catalysts—particularly those functionalized with imidazolium ionomers—can achieve ethanol formation with up to 77.4% FE in a flow cell. That study also showed nonfunctionalized MoP catalysts to be selective for methanol and ethanol with a FE of 21.8%, and confirmed the generation of CO, CH_4_, and C_2_H_4_ via GC and differential electrochemical mass spectrometry (DEMS). These findings strongly suggest that the unquantified portion of FE in our system corresponds to gaseous products, especially under more negative potentials where hydrogen and CO evolution typically dominate.

**Figure 5:**
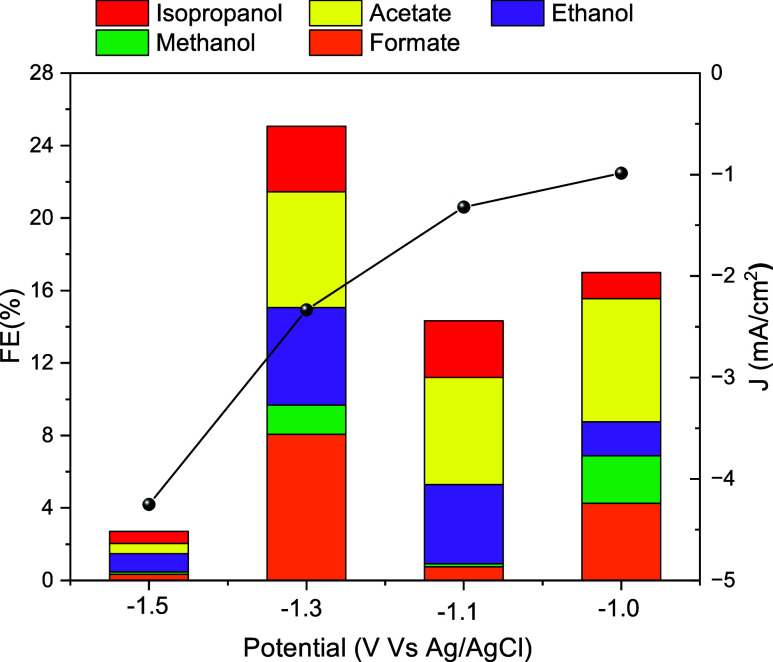
Faradaic efficiency of liquid products (stacked bars) and current density (black spheres) at selected applied potentials.

### In situ EXAFS Experiments

Figures 6 and S6 present a comparison of the XANES and its first derivative vs energy for MoP at different applied potentials in 1 M KOH with CO_2_ saturation and under OCV conditions. The dry and OCV state edge position is ∼20006 eV and marked with a dashed line in Figure 6a, which is consistent with a metallic state for Mo at OCV. At applied potential, the Mo K-edge shows a small shift to higher energies (Figure 6a) but remains metallic (Figure S6).

**Figure 6:**
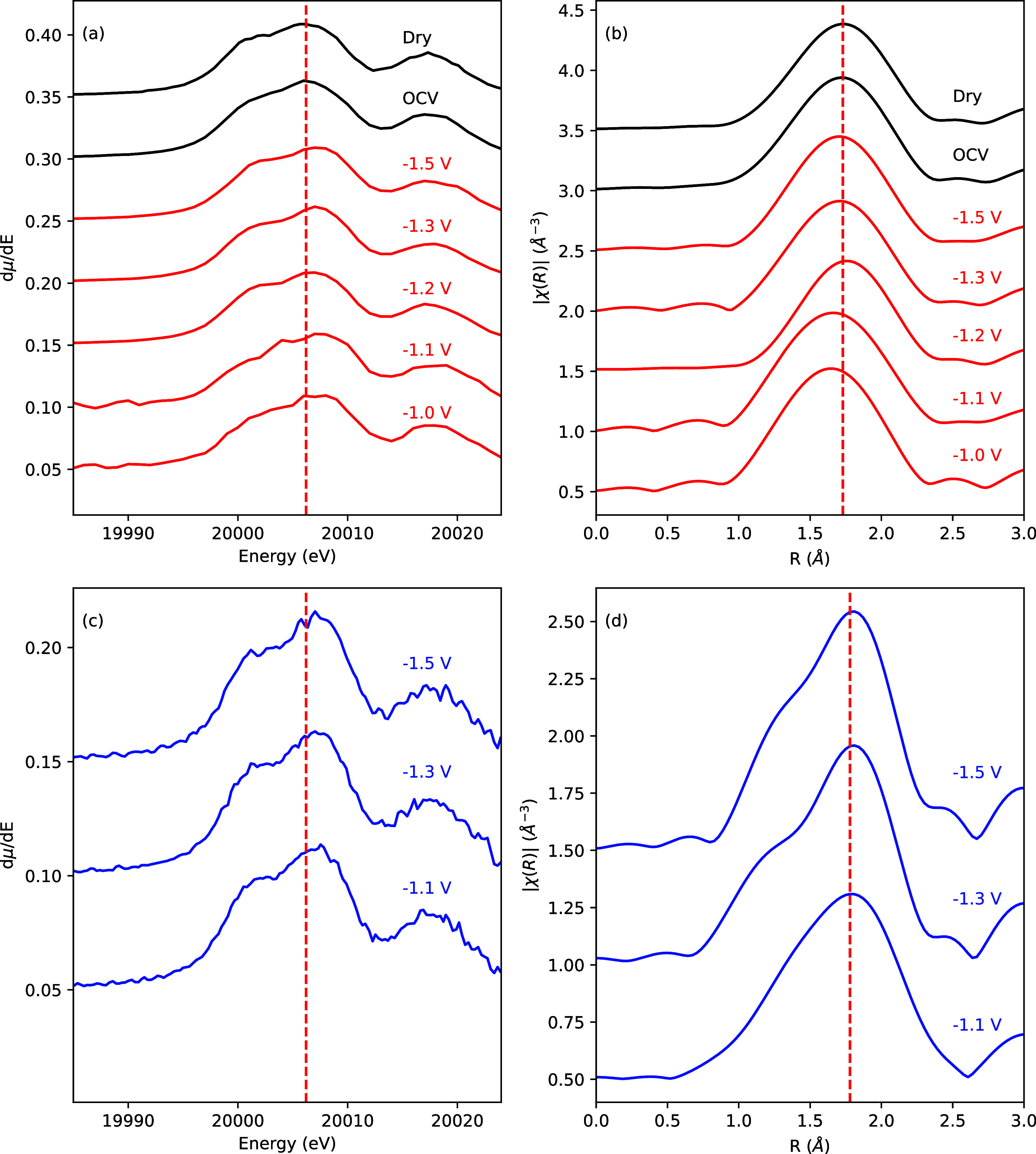
(a) Mo K-edge first-derivative XANES spectra under eCO_2_RR. (b) EXAFS Fourier transforms under eCO_2_RR. (c) XANES under HER. (d) EXAFS Fourier transforms under HER. Red, CO_2_RR; blue, HER.

A cursory analysis of EXAFS Fourier Transform (Figure 6b), shows small changes in the R distance for the first shell. The fit results are summarized in Table 1. Initial observation of Figure 7 suggests that the Mo absorbing atoms experience a slight change in their surrounding environment as the Mo–P bond distance decreases compared to dry conditions when the potential is applied during CO_2_RR. However, as the negative potential increases, the bond distance begins to increase, eventually reaching the same value as at OCV and at −1.3 V during HER. No significant changes in the Mo–P bond distance for HER testing conditions are observed, as can be seen in Figures 6d and 7. While the absolute changes in the Mo–P bond lengths are small, they represent an average over all the Mo atoms in the ∼22 nm nanoparticles and thus are either indicative of a collective change of all Mo–P bonds due to interactions at the nanoparticle surface or a much larger change in the Mo–P bonds at the nanoparticle surface. Indeed, in the extreme cases of spherical particles of 7 and 22 nm, only 26% and 8% respectively of the Mo atoms are on the surface and the average 0.02 Å bond length change would correspond to a change of between 0.08 Å and 0.24 Å in the surface Mo–P bonds.

**Table 1:** Mo–P Radial Distance (*R*) and Debye–Waller Factor (σ^2^) from Mo K-Edge EXAFS Fits for the dry Electrode and In Situ Measurements under CO_2_RR and HER Conditions in 1 M KOH^a^

	**CO**_2_ **RR**		**HER**	
**V vs Ag/AgCl**	*R*_Mo–P_ (Å)	σ^2^ (Å^2^)	*R*_Mo–P_ (Å)	σ^2^ (Å^2^)
Dry	2.397 ± 0.003	0.0085 ± 0.0005	–	–
OCV	2.395 ± 0.004	0.0084 ± 0.0007	–	–
–1.0	2.374 ± 0.010	0.0071 ± 0.0017	–	–
–1.1	2.386 ± 0.015	0.0076 ± 0.0014	2.392 ± 0.005	0.0077 ± 0.0009
–1.2	2.402 ± 0.004	0.0076 ± 0.0008	–	–
–1.3	2.394 ± 0.004	0.0078 ± 0.0007	2.392 ± 0.005	0.0077 ± 0.0009
–1.5	2.392 ± 0.007	0.0084 ± 0.0012	2.392 ± 0.005	0.0068 ± 0.0009

a Global parameters *N*_Mo_–_P_ = 5.8 ± 0.2 and Δ*E*_0_ = 0.3 ± 0.3 were constrained and fitted simultaneously for all data sets.

**Figure 7:**
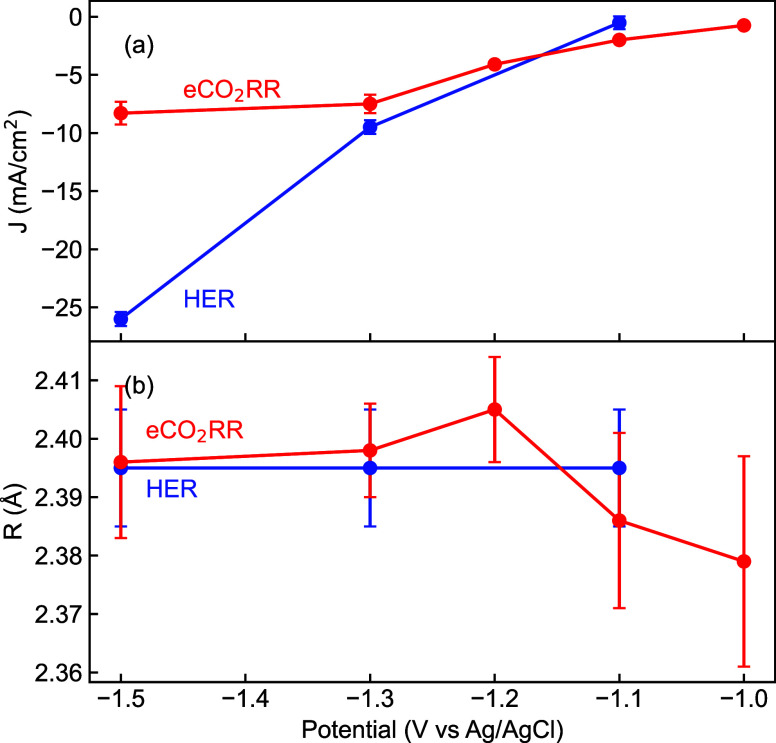
(a) Current density and (b) Mo–P bond distances measured vs applied potential for CO_2_ reduction (red) and hydrogen evolution (blue) reactions.

These observed changes in Mo–P bond distance reflect a dynamically perturbed coordination environment under applied potential. Specifically, under CO_2_RR conditions, these variations may be associated with interactions between Mo active sites and adsorbed intermediates, particularly *CO-related species, which influence the local electronic structure and bonding environment. Previous reported in situ Raman studies of MoP systems showed the role of interfacial effects in stabilizing reaction intermediates.

These observations correlate with selectivity results observed in Figure 5, where at −1.0 V the liquid products FE is relatively low, with acetate and ethanol as the dominant products, and EXAFS data shows slight decrease in the Mo–P bond length, indicating early stage polarization and product intermediate binding. At −1.1 V, the FE, bond length and Debye–Waller factor increase, suggesting enhanced surface restructuring and polarization that increases eCO_2_RR. The highest FE is observed at −1.3 V, which may indicate stronger adsorption of intermediates. However, at −1.5 V, although the current density continues to increase, FE and product selectivity decreases, and as seen in Figure 7 there is a stabilization in the bond distance, indicating a transition toward HER-dominated conditions where active sites may be saturated.

## Conclusions

The synthesized MoP catalyst exhibited increasing activity with the highest FE of 11.75% for C_2+_ products (ethanol and acetate) at −1.3 V vs Ag/AgCl. In situ XANES analysis indicates that the Mo atoms are in a metallic environment with a slight shift in the MoP K-edge upon application of potential, regardless of the CO_2_RR or HER reaction conditions. The EXAFS analysis of the Mo local environment shows a decrease in the average Mo–P bond distance during CO_2_RR activity. While this change is small, it is indicative of either a collective bond reduction or a more significant change of Mo–P bonds at the surface during CO_2_RR. In contrast, when HER dominates or when HER is studied independently, no significant changes in the Mo–P bond distance are observed. Although the magnitude of these variations is modest, they indicate a potential-dependent structural response of MoP under CO_2_ reduction conditions. In conclusion, the comparative analysis of CO_2_RR and hydrogen evolution reaction (HER) conditions reveals that the observed structural changes in MoP are specific to CO_2_ reduction, underscoring the value of in situ X-ray absorption spectroscopy for distinguishing catalyst responses under competing electrochemical pathways.

## Supplementary Material



## Data Availability

The data supporting the findings of this study are provided in the Supporting Information, including ^1^H NMR spectra and quantitative analysis, as well as in situ X-ray absorption spectroscopy data. Additional raw data are available from the corresponding author upon reasonable request.

## Notes

^1^H NMR spectra and quantitative analysis of liquid products, as well as in situ X-ray absorption spectroscopy data (PDF)
