# Supplementary material for: In Situ EXAFS Study of Mo–P Bond Dynamics in Molybdenum Phosphide during CO2 Reduction and Hydrogen Evolution
Source: ACS Omega. 2026 May 15;11(21):31570–6. doi: 10.1021/acsomega.6c02246 (PMC13234655; doi:10.1021/acsomega.6c02246)
Supplement: Supplementary file 1 [file ao6c02246_si_001.pdf]

Supporting Information for
In Situ EXAFS Study of Mo–P Bond Dynamics in Molybdenum Phosphide during CO_2 Reduction and Hydrogen Evolution

Karen A. Castañeda[1,2], Ishani Senevirathna[1,2], Benard Patawah[1,2], Ning Su[1,2], Mohammad Asadi[3], and Carlo U. Segre[1,2]

[1]Department of Physics, Illinois Institute of Technology, 3101 South Dearborn Street, Chicago, IL 60616, USA
[2]Center for Synchrotron Radiation Research and Instrumentation, 3101 South Dearborn Street, Chicago, IL 60616, USA
[3]Department of Chemical and Biological Engineering, Illinois Institute of Technology, 10 West 33rd Street, Chicago, IL 60616, USA

Contents

1 Context S4

2 Nerst equation S4

3 Faradaic Efficiency and Liquid Product Quantification via ^{1}H NMR S4

List of Tables

S1 Electrochemical half-reactions in alkaline media. S11
S2 ^{1}H NMR characteristics of common CO_2RR liquid products. S11
S3 NMR integrals and corresponding concentrations of liquid products at different applied potentials. S11
S4 Faradaic efficiencies (FE) of liquid products obtained at different applied potentials. S12

List of Figures

S1 Chronoamperometry of MoP for 1 h in CO_2-saturated 1 M KOH at various applied potentials for Faradaic efficiency calculations. Current fluctuations arise from gas bubble formation and detachment during electrolysis. S5
S2 ^{1}H NMR analyses for CO_2RR at different applied potentials versus Ag/AgCl. Panels (a,b) correspond to −1.0 V, (c,d) to −1.1 V, (e,f) to −1.3 V, and (g,h) to −1.5 V. Left panels show raw spectra, while right panels show peak deconvolution used to resolve overlapping ethanol and isopropanol signals. Spectra were recorded at 500 MHz using water suppression in 90% electrolyte solution and 10% D_2O, with DMSO as an internal standard. S6
S3 Chronoamperometry of MoP for 20 min in CO_2-saturated 1 M KOH during *in situ* EXAFS experiments. S7
S4 Mo K-edge *in situ* EXAFS under CO_2RR conditions (top to bottom): magnitude of the Fourier transform, real part, and k^2-weighted EXAFS. S8
S5 Mo K-edge *in situ* EXAFS under HER and open-circuit voltage conditions. . S9
S6 Mo K-edge XANES spectra collected under CO_2RR and HER conditions at various applied potentials. S10

1 Context

This document contains supplemental data and analysis supporting the main manuscript titled In Situ *EXAFS Study of Mo–P Bond Dynamics in Molybdenum Phosphide during CO_2 Reduction and Hydrogen Evolution.* Additional experimental results and spectral data are provided to enhance clarity.

2 Nerst equation

All potentials can be converted to be relative to RHE using the Nerst equation

$$V_{\mathrm{RHE}} = V_{\mathrm{Ag/AgCl}} + E^0_{Ag/AgCl-RHE} + 0.0592 \times \mathrm{pH} \tag{S1}$$

where $E^0_{Ag/AgCl-RHE} = 0.210$ V , pH = 9 for CO_2 saturation, and pH = 14 without CO_2 saturation.

3 Faradaic Efficiency and Liquid Product Quantification via ^{1}H NMR

Faradaic efficiency (FE) was used to evaluate the selectivity of a catalyst toward a specific product during CO_2 electroreduction (CO_2RR). It is defined as the percentage of total charge utilized to form a particular product:

$$\mathrm{FE}\ (\%) = \frac{Q_{\mathrm{product}}}{Q_{\mathrm{total}}} \times 100 \tag{S2}$$

Here, Q_{product} is the charge required to form the product, and Q_{total} is the total charge passed during electrolysis, calculated by integrating the current over time:

$$Q_{\mathrm{total}} = \int_0^t I(t)\, dt \tag{S3}$$

The charge associated with the formation of a product is calculated as:

$$Q_{\mathrm{product}} = z \times n \times F \tag{S4}$$

where z is the number of electrons transferred per molecule of product (see Table S1), n is the number of moles of product formed (see Table S3), and F is the Faraday constant (96,485 C mol^{-1}). Calculated FE values for each product and potential can be seen in S4.

Quantification of Liquid Products by ^{1}H NMR

Liquid-phase products were quantified using ^{1}H nuclear magnetic resonance (NMR) spectroscopy. To suppress the water signal, a presaturation technique was applied. Each NMR sample was prepared by mixing 410 µL of the electrolyzed electrolyte, 40 µL of a dimethyl

sulfoxide (DMSO) internal standard, and 50 µL of D_2O, yielding a total volume of 500 µL. The final concentration of DMSO in the NMR mixture was 0.0008 M.

Product concentrations (C_x) were determined using the internal standard method according to:

$$C_x = C_{\mathrm{DMSO}} \cdot \frac{I_x/N_x}{I_{\mathrm{DMSO}}/N_{\mathrm{DMSO}}} \tag{S5}$$

where I_x and I_{DMSO} are the integrated peak areas of the product and DMSO signals, respectively, and N_x and N_{DMSO} are the number of protons contributing to those peaks (see Table S2).

The total amount of each product (n_x) was calculated as:

$$n_x = C_x \cdot \left(\frac{V_{\mathrm{electrolyte}} \cdot V_{\mathrm{NMR}}}{V_{\mathrm{sample}}} \right) \tag{S6}$$

where $V_{\mathrm{electrolyte}}$ is the total electrolyte volume used in the CO_2RR experiment (40 mL), V_{NMR} is the total NMR sample volume (500 µL), and V_{sample} is the volume of the electrolyzed sample added to the NMR tube (410 µL).

Additional Figures

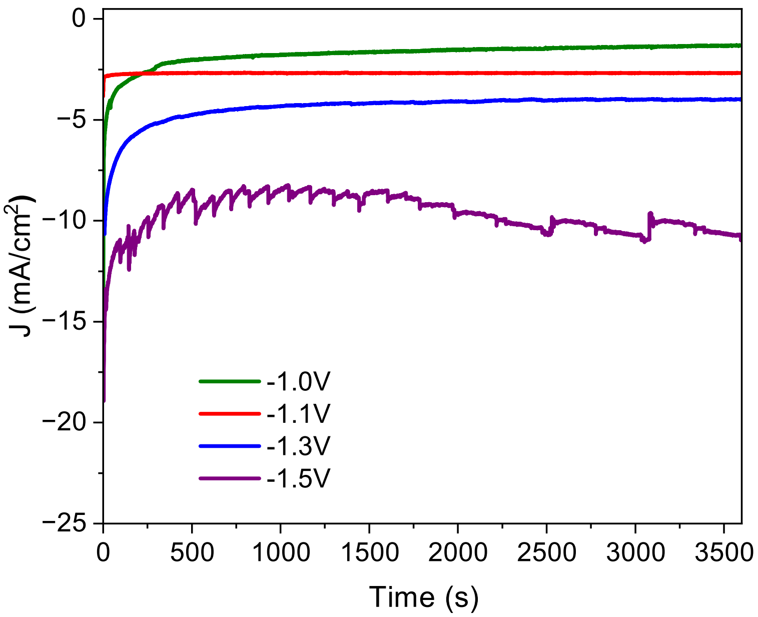

Figure S1: Chronoamperometry of MoP for 1 h in CO_2-saturated 1 M KOH at various applied potentials for Faradaic efficiency calculations. Current fluctuations arise from gas bubble formation and detachment during electrolysis.

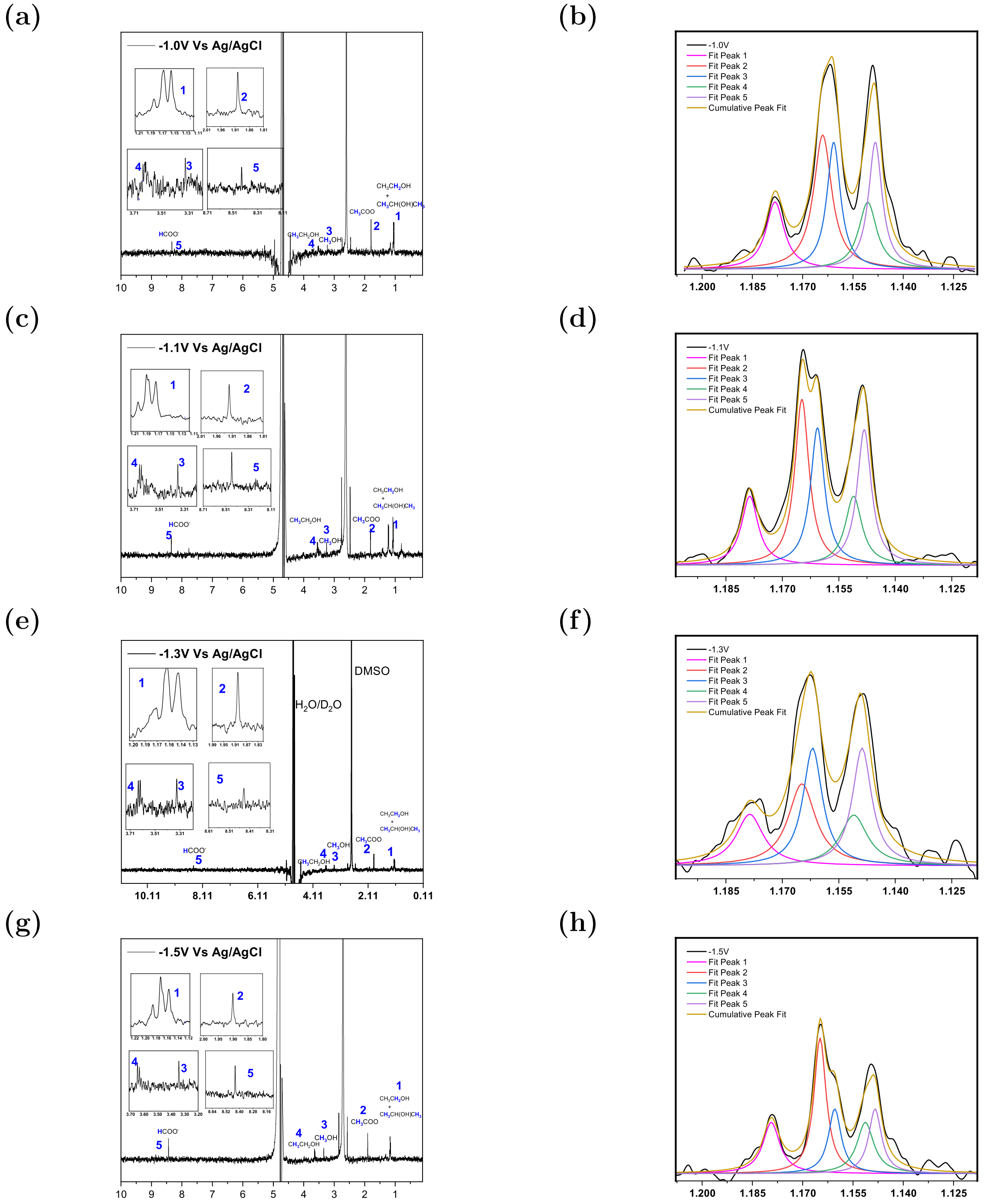

Figure S2: ^{1}H NMR analyses for CO_2RR at different applied potentials versus Ag/AgCl. Panels (a,b) correspond to −1.0 V, (c,d) to −1.1 V, (e,f) to −1.3 V, and (g,h) to −1.5 V. Left panels show raw spectra, while right panels show peak deconvolution used to resolve overlapping ethanol and isopropanol signals. Spectra were recorded at 500 MHz using water suppression in 90% electrolyte solution and 10% D_2O, with DMSO as an internal standard.

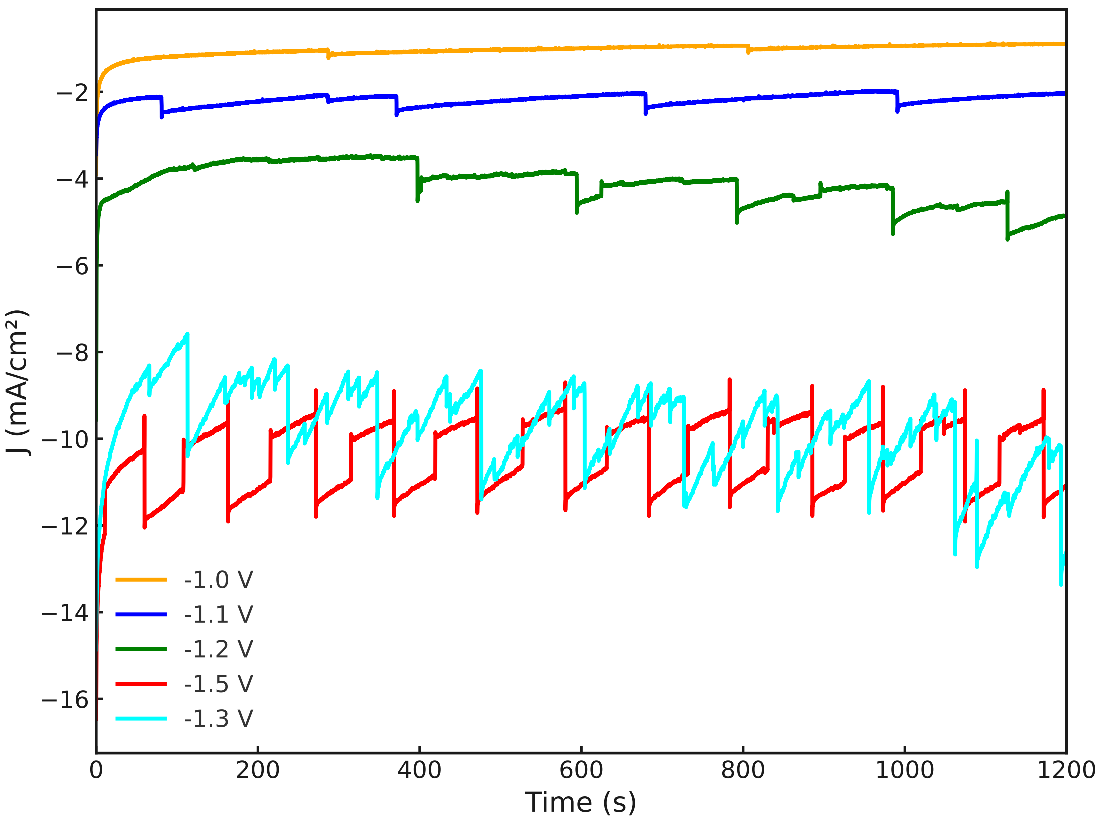

Figure S3: Chronoamperometry of MoP for 20 min in CO_2-saturated 1 M KOH during *in situ* EXAFS experiments.

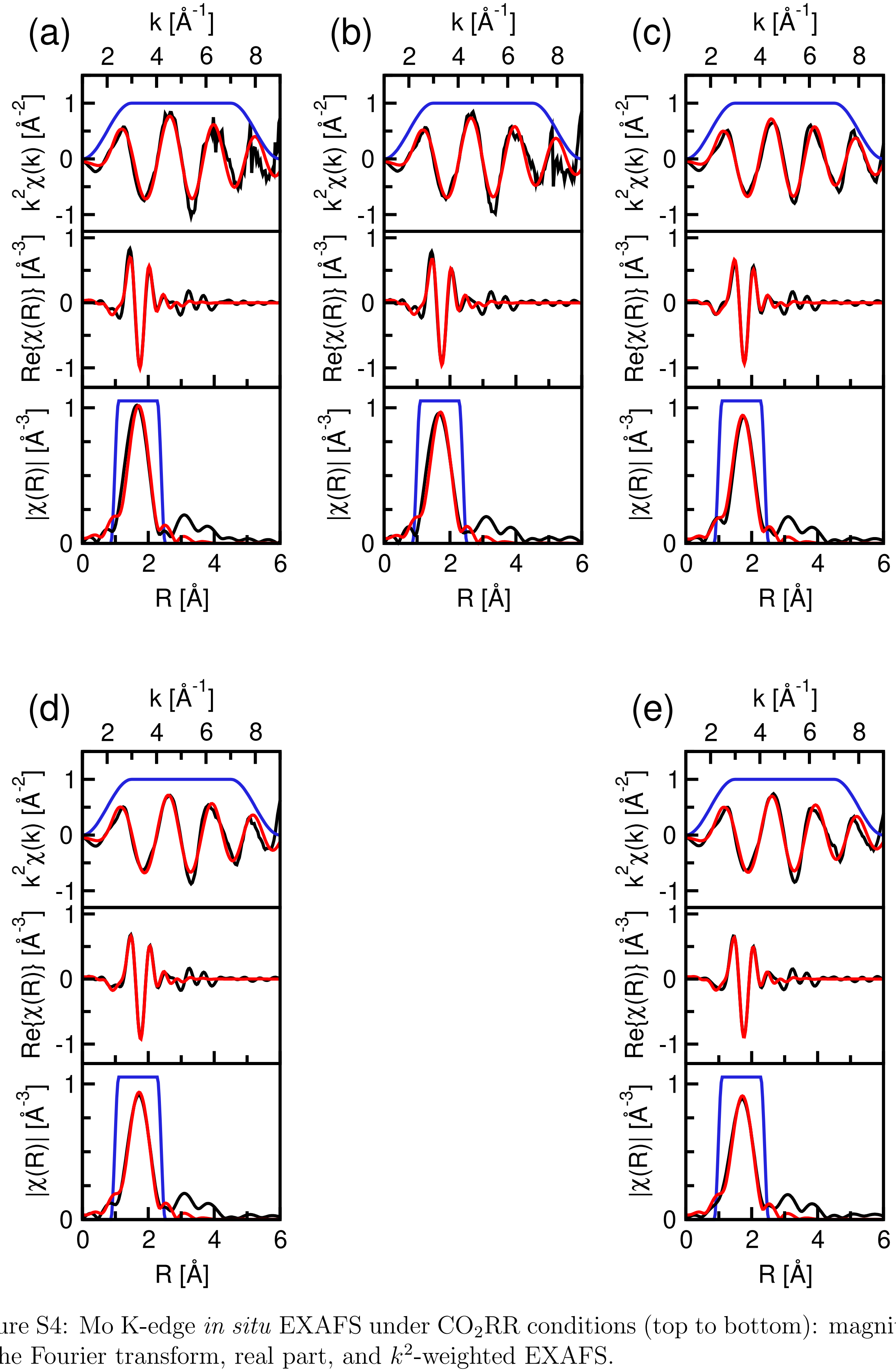

Figure S4: Mo K-edge *in situ* EXAFS under CO_2RR conditions (top to bottom): magnitude of the Fourier transform, real part, and k^2-weighted EXAFS.

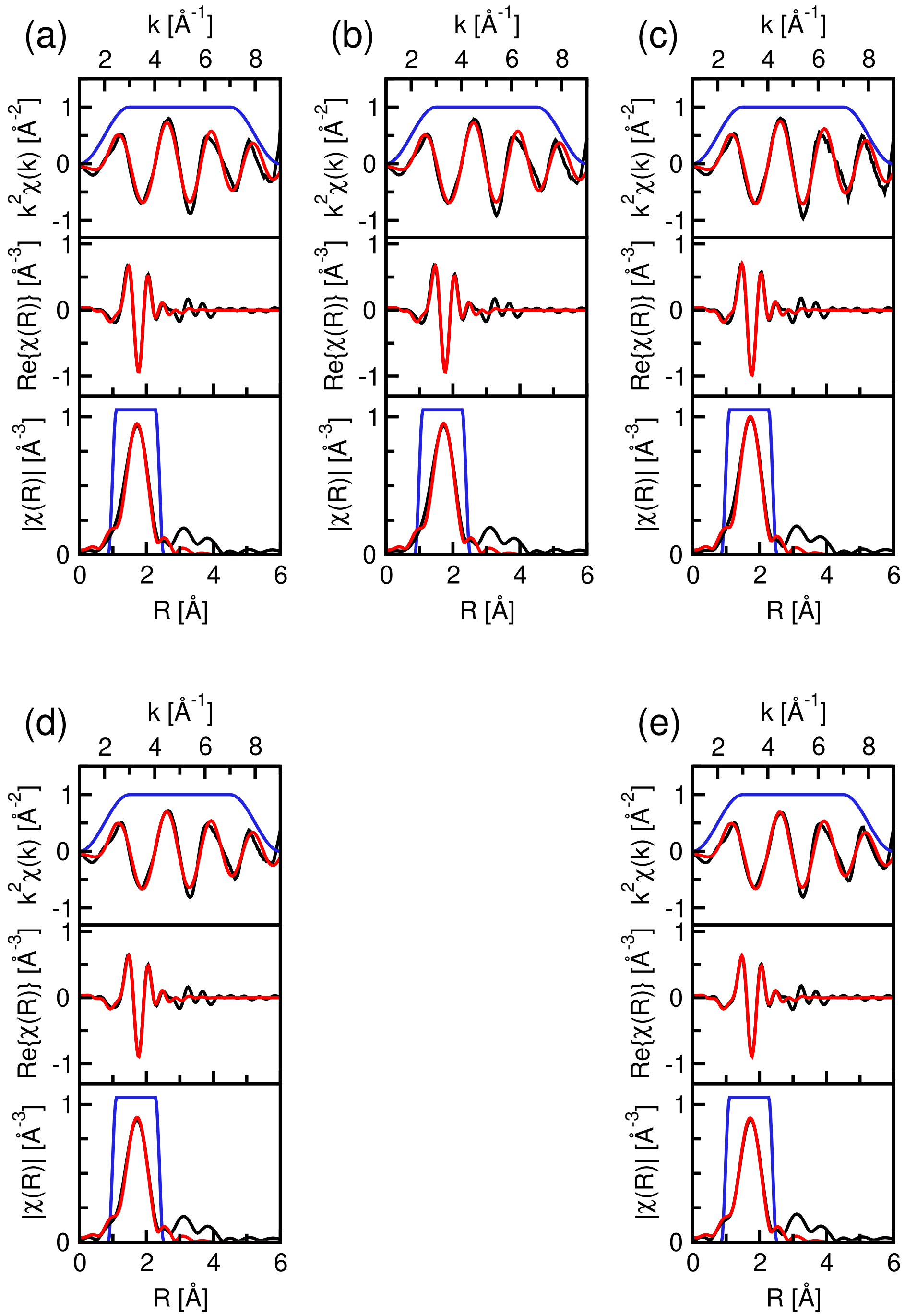

Figure S5: Mo K-edge *in situ* EXAFS under HER and open-circuit voltage conditions.

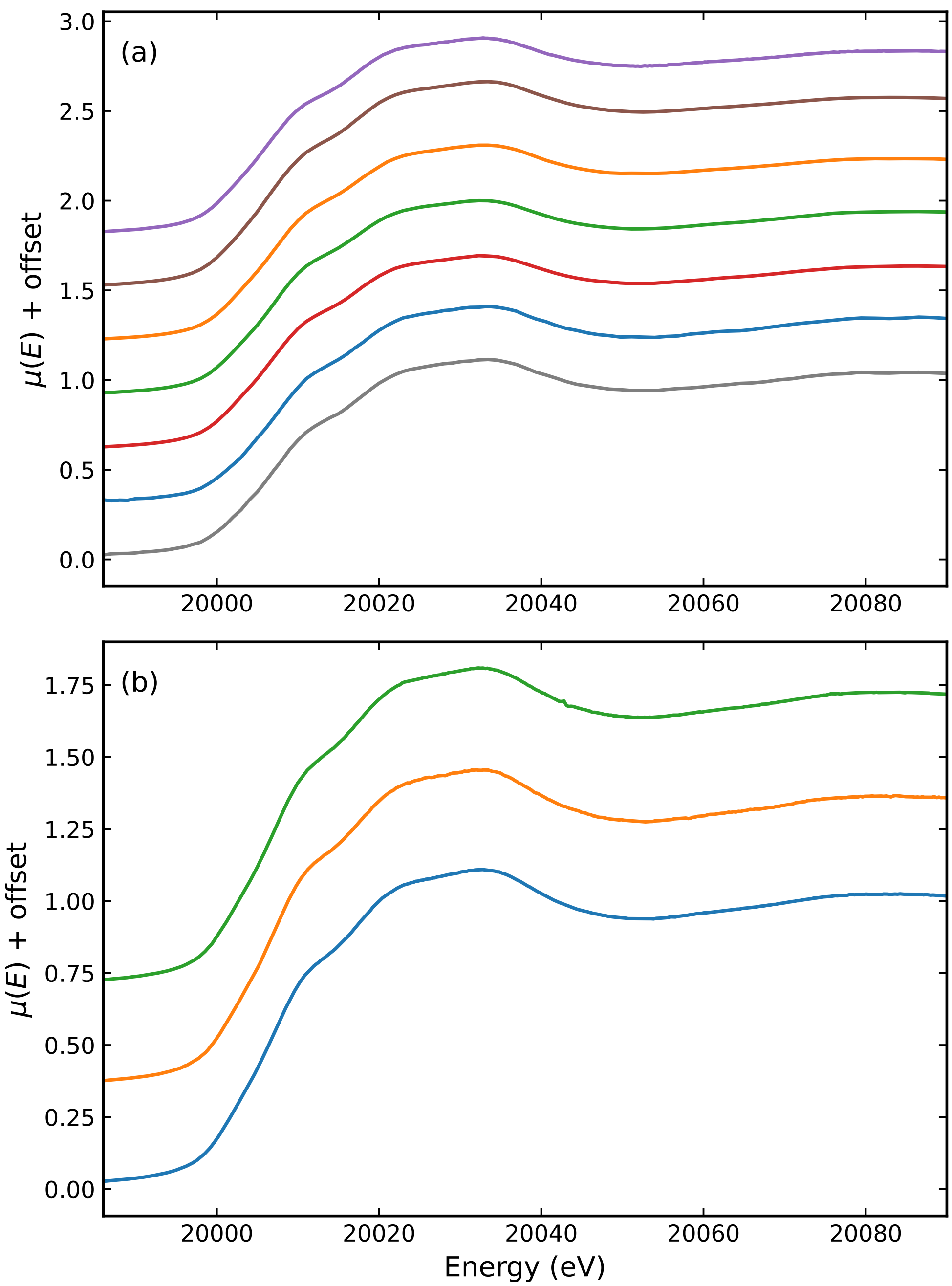

Figure S6: Mo K-edge XANES spectra collected under CO_2RR and HER conditions at various applied potentials.

Additional Tables

Table S1: Electrochemical half-reactions in alkaline media.

**Cathodic half-reactions**		
$2H_2O + 2e^-$	$\rightarrow$	$H_2 + 2OH^-$
$CO_2 + H_2O + 2e^-$	$\rightarrow$	$CO + 2OH^-$
$CO_2 + 2H_2O + 2e^-$	$\rightarrow$	$HCOOH + 2OH^-$
$2CO_2 + 9H_2O + 12e^-$	$\rightarrow$	$CH_3CH_2OH + 12OH^-$
**Anodic half-reaction**		
$2OH^-$	$\rightarrow$	$H_2O + \frac{1}{2}O_2 + 2e^-$

Table S2: ^{1}H NMR characteristics of common CO_2RR liquid products.

Compound	Proton type	# H	Signal type	Chemical shift (ppm)
Ethanol	CH_3	3	Triplet	1.18
Ethanol	CH_2	2	Quartet	3.65
Acetate	CH_3	3	Singlet	1.90
Isopropanol	CH_3	6	Doublet	1.16
Isopropanol	CH	1	Septet	4.01

Table S3: NMR integrals and corresponding concentrations of liquid products at different applied potentials.

**Potential (V)**			**NMR integral**		
	**Methanol**	**Formate**	**Ethanol**	**Isopropanol**	**Acetate**
−1.00	0.00410	0.00670	0.00140	0.00152	0.00800
−1.10	0.00100	0.00160	0.00460	0.00440	0.00930
−1.30	0.00300	0.00500	0.01000	0.00900	0.01780
−1.50	0.00100	0.00220	0.00330	0.00300	0.00290
**Potential (V)**			**Concentration (mol L^{-1})**		
	**Methanol**	**Formate**	**Ethanol**	**Isopropanol**	**Acetate**
−1.00	8.0×10^{-6}	3.92×10^{-5}	2.89×10^{-6}	1.48×10^{-6}	1.56×10^{-5}
−1.10	1.95×10^{-6}	9.40×10^{-6}	8.98×10^{-6}	4.29×10^{-6}	1.81×10^{-5}
−1.30	5.85×10^{-6}	2.93×10^{-5}	1.95×10^{-5}	8.78×10^{-6}	3.47×10^{-5}
−1.50	1.95×10^{-6}	1.29×10^{-5}	6.58×10^{-6}	2.93×10^{-6}	5.65×10^{-6}

NMR integrals are reported relative to the DMSO internal standard, whose signal was normalized to an integral value of 1. Concentrations were calculated using the internal standard method described in the text.

Table S4: Faradaic efficiencies (FE) of liquid products obtained at different applied potentials.

Potential (V vs RHE)	Formate (%)	Methanol (%)	Ethanol (%)	Acetate (%)	Isopropanol (%)
−1.5	0.325	0.148	0.995	0.571	0.664
−1.3	8.068	1.614	5.379	6.383	3.631
−1.1	0.761	0.159	4.376	5.898	3.139
−1.0	4.264	2.609	1.884	6.788	1.451
